# Seasonal surveillance of *Salmonella* prevalence, antimicrobial resistance, and genetic relatedness in chickens from slaughterhouses and retail markets in Northeast Thailand

**DOI:** 10.14202/vetworld.2026.1550-1563

**Published:** 2026-04-24

**Authors:** Zhihui Zhang, Fanan Suksawat, Chaiwat Pulsrikarn, Sunpetch Angkititrakul

**Affiliations:** 1 Faculty of Veterinary Medicine, Khon Kaen University, Khon Kaen, Thailand; 2 National Institute of Health, Department of Medical Sciences, Ministry of Public Health, Nonthaburi, Thailand

**Keywords:** antimicrobial resistance, chicken, food safety, genetic relatedness, prevalence, poultry supply chain, seasonal variation, *Salmonella*

## Abstract

**Background and Aim:**

*Salmonella* is a major foodborne zoonotic pathogen that threatens public health and poultry production worldwide. Although its prevalence and antimicrobial resistance (AMR) in Thai poultry have been widely reported, continuous comparative surveillance across slaughterhouses and retail markets with seasonal stratification remains limited, particularly in Northeast Thailand. This study aimed to systematically compare the prevalence, serotype distribution, AMR profiles, and genetic relatedness of *Salmonella* isolates recovered from chicken slaughterhouses and retail markets, with emphasis on seasonal and supply chain variations.

**Materials and Methods:**

A total of 689 swab samples were collected from two slaughterhouses and two retail markets in Khon Kaen Province, Thailand, across three seasons (summer, rainy, and winter) from March 2023 to February 2024. Isolation and identification of *Salmonella* were performed according to ISO 6579-1:2017. Serotyping was conducted using the White–Kauffmann–Le Minor scheme. Antimicrobial susceptibility testing was performed against 14 antibiotics using the disk diffusion method following Clinical and Laboratory Standards Institute guidelines. Genetic relatedness of selected isolates was assessed using pulsed-field gel electrophoresis. Statistical analyses included chi-square tests, relative risk estimation, and multivariable logistic regression.

**Results:**

The overall prevalence of *Salmonella* was 32.37% (223/689), with significantly higher contamination in retail markets than slaughterhouses. Prevalence peaked during the rainy season (45.29%), whereas multidrug resistance was highest in winter, particularly in slaughterhouse isolates. The predominant serotypes were *Salmonella* Agona (15.25%) and *Salmonella* Saintpaul (14.79%). High resistance rates were observed to streptomycin (43.05%), ampicillin (34.53%), and nalidixic acid (30.49%). Multidrug resistance was detected in 32.43% of isolates. Logistic regression analysis identified retail markets and rainy and winter seasons as independent risk factors for contamination. Pulsed-field gel electrophoresis demonstrated clonal relatedness among isolates within similar locations and seasons, with evidence of cross-contamination along the supply chain.

**Conclusion:**

This study provides a comprehensive, year-round comparative assessment of *Salmonella* contamination and AMR in slaughterhouses and retail markets in Northeast Thailand. The distinct seasonal dissociation between peak prevalence and peak multidrug resistance highlights the need for season-specific and site-targeted surveillance and intervention strategies within the poultry supply chain.

## INTRODUCTION

*Salmonella* is one of the most significant foodborne zoonotic pathogens worldwide, posing a substantial burden on public health and causing significant economic losses in the poultry industry [1, 2]. Human salmonellosis, often characterized by gastroenteritis, fever, and abdominal cramps, is frequently linked to the consumption of contaminated poultry products [3, 4]. In poultry, *Salmonella* can cause both clinical disease and subclinical carrier states, leading to impaired growth, increased mortality, and costly control measures [5].

The widespread use of antimicrobials for therapy, prophylaxis, and growth promotion in animal production has been a key driver in the selection and dissemination of antimicrobial resistance (AMR) [6, 7]. The emergence of multidrug-resistant (MDR) *Salmonella* strains is particularly concerning and constitutes a major public health threat. This resistance is frequently mediated and facilitated by mobile genetic elements, such as plasmids, transposons, and integrons, which enable the horizontal transfer of resistance genes (e.g., *bla*_TEM1_, *aadA1*, *sul1*, *tet (A)*) across bacterial populations [8, 9]. These mechanisms significantly compromise the efficacy of first-line antibiotics in both veterinary and human medicine, representing a critical One Health challenge [10].

In Thailand, national surveillance data consistently identify non-typhoidal *Salmonella* as one of the leading causes of bacterial foodborne illness, with poultry products being a primary environmental and foodborne reservoir. The convergence of high *Salmonella* prevalence in poultry flocks and the emergence of MDR strains in human clinical cases, as highlighted in reports from the Thai Ministry of Public Health and the WHO, underscores the direct and tangible threat that poultry-borne drug-resistant *Salmonella* strains pose to human health and the efficacy of clinical treatment [11, 12]. The sequential transmission of such resistant clones and their genetic determinants through the food chain, from farm to slaughterhouse to retail market, underscores the need for comprehensive integrated surveillance strategies that go beyond phenotypic resistance to fully understand the genetic basis of AMR [13].

In Southeast Asia, including countries such as Thailand, large-scale poultry production and the widespread use of antimicrobial agents have made the region a hotspot for AMR development. However, most existing monitoring studies have notable limitations. Firstly, many studies predominantly focus on isolated segments of the production chain, either farms/slaughterhouses or retail markets, thus lacking continuous, systematic comparisons across the entire post-harvest “slaughterhouse-to-market” distribution chain. This hinders the elucidation of dynamic changes in *Salmonella* contamination and AMR profiles during transmission. Secondly, research on how climatic seasonal factors unique to tropical regions, such as high temperatures and rainy seasons, influence *Salmonella* contamination rates and AMR profiles remains limited. While studies have documented *Salmonella* prevalence across different Thai regions [14, 15], continuous monitoring data covering multiple seasons and systematically comparing both slaughterhouses and retail markets, particularly in Thailand’s Northeast region, remain absent. It is well established that environmental seasonal factors such as temperature and humidity influence bacterial survival and transmission [16], yet this critical factor has not received sufficient attention within regional surveillance systems.

Despite extensive documentation of *Salmonella* prevalence and AMR in poultry production systems, several critical knowledge gaps remain. First, most studies in Thailand and other Southeast Asian countries have focused on isolated points within the poultry production continuum, such as farms, slaughterhouses, or retail markets, without integrating these stages into a unified “slaughterhouse-to-market” framework. This fragmented approach limits the ability to understand transmission dynamics, cross-contamination pathways, and amplification points of *Salmonella* along the supply chain. Second, although seasonal variations in bacterial contamination have been reported, there is a lack of systematic, year-round surveillance that simultaneously evaluates both prevalence and MDR patterns across distinct climatic periods. In particular, the potential dissociation between seasonal peaks of contamination and resistance remains poorly understood.

Furthermore, while phenotypic resistance profiles are widely reported, there is limited incorporation of molecular epidemiological tools to assess the genetic relatedness of circulating *Salmonella* strains across different supply chain nodes. This restricts the ability to identify clonal dissemination and trace contamination sources. Additionally, Northeast Thailand remains underrepresented in national surveillance datasets, despite its significant role in poultry production and distribution. The absence of integrated, region-specific data combining epidemiological, antimicrobial, and genetic perspectives represents a substantial gap in current One Health surveillance efforts. Addressing these limitations is essential for developing targeted, evidence-based intervention strategies tailored to both seasonal risks and specific points within the poultry supply chain.

Therefore, the present study was designed to provide a comprehensive, integrated assessment of *Salmonella* contamination along the poultry supply chain in Northeast Thailand. Specifically, the study aimed to (i) determine and compare the prevalence of *Salmonella* in chicken samples collected from slaughterhouses and retail markets, (ii) evaluate seasonal variations in contamination across summer, rainy, and winter periods, (iii) characterize the serotype distribution of *Salmonella* isolates, (iv) assess AMR patterns, including the prevalence of MDR, and (v) investigate the genetic relatedness of dominant *Salmonella* strains using pulsed-field gel electrophoresis (PFGE).

By integrating epidemiological, phenotypic, and molecular analyses across both spatial (supply chain stages) and temporal (seasonal) dimensions, this study seeks to elucidate critical risk factors driving *Salmonella* contamination and AMR dissemination. The findings are expected to support the development of season-specific and node-targeted surveillance and control strategies, thereby contributing to improved food safety, antimicrobial stewardship, and One Health outcomes in the regional poultry sector.

## MATERIALS AND METHODS

### Ethical approval

The study protocol was reviewed and approved by the Institutional Animal Care and Use Committee of Khon Kaen University, Thailand (approval no. IACUC-KKU-111/67). All procedures involving animals were conducted in strict accordance with national guidelines for animal research and welfare, and in compliance with internationally accepted standards, including the ARRIVE 2.0 guidelines and the principles outlined in the Guide for the Care and Use of Laboratory Animals.

This study was based on non-invasive sampling procedures conducted under routine surveillance conditions. Cloacal swab samples were collected by trained veterinarians using standardized handling techniques to minimize stress, discomfort, and potential injury to the birds. No experimental infection, invasive intervention, or euthanasia was performed as part of this study. All sampling procedures adhered to the principles of replacement, reduction, and refinement (3Rs), ensuring that the number of animals involved was limited to the minimum required to achieve scientific validity while maintaining high standards of animal welfare.

The chickens included in this study originated from commercial farms operating under the Thai Agricultural Standard TAS 6901-2017 and were handled within standard production and processing environments. Permission to access animals and collect samples at slaughterhouses and retail markets was obtained from facility operators and relevant authorities prior to sampling. All sampling activities were carried out in a manner that did not interfere with routine farm or slaughterhouse operations.

Biosafety and biosecurity measures were strictly implemented throughout the study. All laboratory procedures involving *Salmonella* were conducted in a biosafety level 2 laboratory in accordance with institutional biosafety regulations. Personnel were trained in microbiological safety practices, and appropriate personal protective equipment was used at all times to prevent occupational exposure and environmental contamination.

Overall, the study design ensured full compliance with ethical standards for animal use in research while maintaining scientific rigor and data reliability.

### Study period and location

This study was a cross-sectional seasonal surveillance study conducted along the poultry supply chain in Khon Kaen Province, Thailand. Over one year (March 2023 to February 2024), we simultaneously monitored and compared *Salmonella* contamination at two critical nodes, slaughterhouses and retail markets, across three climatic seasons (summer, rainy, and winter). The comparative design aimed to identify site-specific and season-specific risk patterns within the local poultry production and retail system.

### Description of slaughterhouses and markets

The chickens sampled in this study originated from standardized commercial farms operating under the Thai Agricultural Standard TAS 6901-2017, which mandates veterinary oversight and restricts the use of certain critically important antimicrobials, and which had been approved by the Department of Livestock Development. All broilers were of a mixed breed, aged 35–40 days, and sourced from a single integrated production company. This common origin ensures a consistent baseline of farm management and biosecurity. Birds were transported to the slaughterhouses in dedicated vehicles over a short travel duration (1–2 h). The two participating slaughterhouses were small scale, manually operated facilities processing approximately 500–800 birds per day, utilizing municipal water and implementing basic daily cleaning routines. Following processing, carcasses were distributed to the two traditional wet markets included in the study, where they were displayed and sold at ambient temperature on open stalls, with no dedicated cold chain facilities.

### Sampling strategy and seasonal classification

A total of 689 swabs (including cloacal swab and carcass swab) were collected during different seasons from slaughterhouses (n = 2) and markets (n = 2) in Khon Kaen Province, Northeast Thailand. Three seasons were defined as summer (March–May, 32°C–38°C, humidity rate 66%–82%), rainy (June–October, 27°C–30°C, humidity rate 82%–88%), and winter (November–February, 20°C–28°C, humidity rate 50%–75%). The sampling period spanned one year (March 2023 to February 2024), covering a temperature–humidity index range of 65–80, representing 16 discrete intervals.

### Sample size determination

A systematic, repeated sampling strategy was employed: the same four sites were sampled once per quarter across all three seasons. Based on a reported *Salmonella* prevalence of 23.3% in chicken slaughterhouses [6], the sample size was calculated using the formula n = Z² × P × (1 − P) / e², with a 95% confidence interval and 5% margin of error. Using Z = 1.96, e = 0.05, and p = 0.233, the required sample size was 228. For markets, with a reported prevalence of 21.33% [17], the required sample size was 258. The total sample collected in this study was 689, which exceeded the minimum calculated sample size. The number of samples collected per season and site is shown in Table 1.

**Table 1:** Number of samples collected from slaughterhouses and markets across different seasons.

Season	Sampling site	Number of samples	Sample type
Summer	Slaughterhouse 1	57	Cloacal swab
Summer	Slaughterhouse 2	44	Cloacal swab
Summer	Market 1	34	Carcass swab
Summer	Market 2	33	Carcass swab
Rainy	Slaughterhouse 1	78	Cloacal swab
Rainy	Slaughterhouse 2	88	Cloacal swab
Rainy	Market 1	71	Carcass swab
Rainy	Market 2	55	Carcass swab
Winter	Slaughterhouse 1	100	Cloacal swab
Winter	Slaughterhouse 2	66	Cloacal swab
Winter	Market 1	49	Carcass swab
Winter	Market 2	77	Carcass swab
Total		689	

### Isolation and identification of *Salmonella*

Isolation and identification of *Salmonella* were performed according to ISO 6579-1:2017 [18]. After collection, swabs stored in transfer medium were placed in Buffered Peptone Water (BPW) for pre-enrichment and incubated at 37°C for 24 h. Subsequently, three loopfuls of the culture were transferred to Modified Semisolid Rappaport Vassiliadis Medium (MSRV) plates for selective enrichment and incubated at 42°C for 24 h. Positive colonies were streaked onto Xylose Lysine Deoxycholate agar (XLD) plates and incubated for 24 h [19]. Presumptive *Salmonella* colonies were confirmed using Triple Sugar Iron (TSI, Difco) and Motility Indole Lysine (MIL, Difco) tests. All the above media and reagents were obtained from Difco Laboratories, Detroit, MI, USA. All isolates were stored in Luria–Bertani (LB) medium containing 20% glycerol at −80°C.

### Serotyping of *Salmonella* isolates

Serological identification was performed using diagnostic polyvalent and monovalent *Salmonella* “O” and “H” antisera (Denka Seiken, Tokyo, Japan). At least one isolate per positive sample was selected for serotyping. Isolates were subsequently stored in tryptic soy broth (Difco Laboratories) containing 15% glycerol at −80°C.

### Antimicrobial susceptibility testing

The panel included agents with reported usage in Thai poultry (e.g., ampicillin, tetracycline, streptomycin) alongside representatives of the WHO highest-priority critically important antimicrobials (HPCIA) (e.g., fluoroquinolones, third-generation cephalosporins, carbapenems). Colistin was excluded based on its restricted use in Thai food animals following the 2017 national ban on antimicrobial growth promoters.

Antimicrobial susceptibility testing was performed against 14 antibiotics using the disk diffusion method on Mueller–Hinton agar (Difco), in accordance with Clinical and Laboratory Standards Institute (CLSI) guidelines [20]. The antimicrobial agents (Oxoid, Liofilchem, UK) tested included streptomycin (S, 10 μg), norfloxacin (NOR, 10 μg), tetracycline (TE, 30 μg), gentamicin (GM, 10 μg), sulfamethoxazole/trimethoprim (SXT, 23.75 μg), imipenem (IPM, 10 μg), cefotaxime (CRO, 30 μg), chloramphenicol (C, 30 μg), ciprofloxacin (CIP, 5 μg), nalidixic acid (NA, 30 μg), ampicillin (AM, 10 μg), ceftazidime (CAZ, 30 μg), ceftriaxone (CTX, 30 μg), and amoxicillin/clavulanic acid (AMC, 20/10 μg). Escherichia coli ATCC 25922 (ATCC, Manassas, VA, USA) was used for quality control. Results were accepted only when the zone diameters for the quality control strain fell within CLSI-specified reference ranges. Multidrug resistance was defined as resistance to agents from three or more distinct antibiotic classes [21].

### PFGE and cluster analysis

To investigate the genetic relatedness of the dominant *Salmonella* population, PFGE was performed on a subset of isolates comprising all available strains of the most prevalent serotype, *S*. Agona (n = 34). PFGE was performed according to the PulseNet standard protocol [22], using CHEF DRIII (Bio-Rad, Hercules, CA, USA). Briefly, isolates were grown on Nutrient Agar at 37°C for 16 h. Bacterial cells were suspended in TE buffer (100 mM Tris-HCl, 100 mM EDTA, pH 8.0) and adjusted to an OD610 of 1.4. The cells were embedded in agarose plugs and lysed in lysis buffer (50 mM Tris-HCl, 50 mM EDTA, 1% Sarkosyl, 1 mg/mL proteinase K) at 55°C for 16-20 h. Plugs were washed 4 times with TE buffer and digested with 50 U of XbaI (New England Biolabs) at 37°C overnight. PFGE was performed in 1% agarose gel using 0.5× TBE buffer at 14°C for 18 h [23]. Plug slices of 3–5 mm width were placed in a 1.5 mL microcentrifuge tube containing 200 μL of 1× restriction buffer (NE Buffer supplemented with 50 U of XbaI-digested restriction enzyme) and incubated overnight at 37°C. Plug slices were washed for 30 min with 0.5× TBE buffer and subsequently loaded into the wells of a 1% agarose gel, and the wells were overlaid with 1% agarose dissolved in 0.5× TBE. After solidification, the gel was electrophoresed for 18 h.

PFGE profiles were analyzed using BioNumerics software (version 4.5, Bio-Rad). Dendrograms were constructed using the unweighted pair group method with arithmetic mean, with the Dice similarity coefficient, optimization set to 1%, and tolerance set to 1%. Isolates with 100% similarity were considered to belong to the same PFGE type.

### Statistical analysis

Data were processed using Excel, SPSS 20.0 (IBM Corp., Armonk, NY, USA), and GraphPad Prism 10.1.3. Categorical data were analyzed using the chi-square test, with statistical significance set at p < 0.05 and high statistical significance at p < 0.01.

The prevalence of *Salmonella* was calculated for each season, and differences between seasons were evaluated by the chi-square test (χ²). Relative risk (RR) was used to assess the likelihood of *Salmonella* occurrence associated with seasonal factors. In addition, the independent effects of season and location type were assessed using multivariable logistic regression. Results are reported as adjusted odds ratios (aOR) with 95% confidence intervals (CI).

## RESULTS

### *Salmonella* prevalence in slaughterhouses and markets with seasonal changes

This study found an overall *Salmonella* prevalence of 32.37% (223/689), but its distribution showed significant spatial heterogeneity and seasonal clustering. Spatially, contamination levels varied significantly across sampling sites (χ² = 25.48, p < 0.0001). The overall prevalence at retail markets (particularly Market-1, 60.83%) was significantly higher than that at slaughterhouses (18.23%–22.97%). Seasonally, the distribution of positive samples was generally non-uniform within most individual sites (most p_1_ < 0.05). Overall, positive samples were predominantly concentrated in the rainy (45.29%) and winter (37.22%) seasons, which together accounted for more than 82% of total detections. Further testing indicated that this seasonal clustering pattern was particularly pronounced in winter and differed significantly among sampling sites (p_2_ < 0.0001). These results collectively demonstrate that *Salmonella* contamination status is significantly influenced by both the “sampling site” and “season” (Table 2).

**Table 2:** Seasonal distribution of slaughterhouses and markets from chicken.

Source	Numbers	Positive (%)	Summer (%)	Rainy (%)	Winter (%)	χ²	P1 value
Slaughterhouse 1	235	54 (22.97)	7 (2.99)	29 (12.34)	18 (7.66)	15.15	0.0005
Slaughterhouse 2	181	33 (18.23)	6 (3.31)	20 (11.05)	7 (3.87)	10.12	0.0006
Market 1	120	73 (60.83)	11 (9.16)	25 (20.83)	37 (30.83)	15.70	0.0004
Market 2	153	63 (41.18)	15 (9.80)	27 (17.65)	21 (13.74)	3.43	0.18 (ns)
Total	689	223 (32.37)	39 (17.49)	101 (45.29)	83 (37.22)	–	–
χ² (spatial/seasonal)	–	25.48	4.90	9.25	28.27	–	–
P2 value	–	<0.0001	0.175 (ns)	0.026	<0.0001	–	–

P1 values represent chi-square goodness-of-fit tests for seasonal distribution uniformity of positive isolates within each sampling site. P2 values indicate chi-square goodness-of-fit tests for spatial distribution uniformity of positive isolates across sampling sites within each seasonal category.

To quantify the contamination risk across seasons, this study calculated the RR for the rainy and winter seasons, using summer as the reference. The analysis was stratified by sample type, revealing distinct seasonal risk patterns depending on the sample source. For anal swabs (reflecting animal carriage), the risk of *Salmonella* detection in the rainy season was 2.29 times higher than in summer, a statistically significant increase. In contrast, the risk in winter was not significantly different from that in summer. Conversely, for carcass swabs (reflecting processing contamination), although the detection rates in the rainy and winter seasons were numerically higher than those in summer, neither RR reached statistical significance. This indicates that the influence of seasonal factors on *Salmonella* prevalence is primarily evident at the farm/transport stage (animal carriage), while its effect may be masked or mitigated at the processing stage, likely by other factors such as slaughterhouse hygiene practices (Table 3).

**Table 3:** *Salmonella* prevalence and relative risk (RR) among different seasons.

Sample category	Season	Total	Positive	Prevalence (%)	RR	95% CI	p-value
	Summer	101	13	12.87	1(ref)	–	–
Anal swab	Rainy	166	49	29.52	2.29	1.32–4.00	0.003
	Winter	166	25	15.06	1.17	0.63–2.17	0.617
	Summer	67	26	38.81	1(ref)	–	–
Carcass swab	Rainy	126	52	41.27	1.06	0.75–1.51	0.732
	Winter	126	58	46.03	1.19	0.86–1.65	0.299

RR uses summer as the reference group; 95% CI denotes 95% confidence interval; P-values are calculated based on chi-square test or Fisher’s exact test.

To assess the independent effects of each risk factor, this study further constructed a multivariate logistic regression model. After adjusting for the confounding effects of season and sampling location, the analysis revealed that retail markets (compared to slaughterhouses) and the rainy season and winter (compared to summer) each represented independent risk factors for *Salmonella* contamination (Table 4). Specifically, the contamination risk for market samples was 4.82 times higher than that for slaughterhouse samples (aOR = 4.82, 95% CI: 3.42–6.79); while the risks during the rainy season and winter were 3.81 times (aOR = 3.81, 95% CI: 2.40–6.04) and 2.80 times (aOR = 2.80, 95% CI: 1.73–4.53) higher than in summer, respectively. The model demonstrated good fit (Hosmer-Lemeshow test, p = 0.62). Collectively, the univariate and multivariable analyses demonstrate that *Salmonella* contamination is independently and statistically significantly associated with both spatial (retail market location) and temporal (rainy and winter seasons) factors, with market samples and the rainy season posing the highest respective contamination risks.

**Table 4:** Multivariable logistic regression analysis of independent risk factors for *Salmonella* contamination in chickens.

Factor	Category	Adjusted odds ratio	95% Confidence interval	p-value
Location type	Slaughterhouse (Ref)	1	–	–
	Market	4.82	3.42 – 6.79	<0.001
	Summer (Ref)	1	–	–
Season	Rainy	3.81	2.40 – 6.04	<0.001
	Winter	2.80	1.73 – 4.53	<0.001

Model fit statistics: Hosmer-Lemeshow goodness-of-fit test, χ² = 5.32, p = 0.62; Nagelkerke R² = 0.28. The overall model was statistically significant (*p* < 0.001).

### Serotype distribution from slaughterhouses and markets

Serotyping of 223 *Salmonella* isolates revealed that *Salmonella* Agona (15.25%) and *Salmonella* Saintpaul (14.79%) were the most predominant serotypes, while *Salmonella* Singapore (10.31%) was also widely distributed across all sampling sites. The serotype distribution exhibited significant seasonal variation: although the rainy season accounted for the highest total number of isolates (68.16%), several important serotypes, including *S*. Agona, *S*. Bareilly, and *S*. Rissen, were predominantly detected during the winter season (Table 5).

**Table 5:** Serotype distribution of different isolates from slaughterhouses and markets.

*Salmonella* Group	Serotype	Slaughterhouse 1	Slaughterhouse 2	Market 1	Market 2	Season	Total
	*Salmonella* Saintpaul	17 (26.98)	14 (22.58)	2 (3.85)	0	Rainy	33 (14.79)
	*Salmonella* Agona	13 (20.63)	17 (27.42)	2 (3.85)	2 (4.76)	Winter	34 (15.25)
	*Salmonella* Stanley	1 (0.16)	0	8 (15.38)	2 (4.76)	Rainy	11 (0.49)
B	*Salmonella* enterica subsp. enterica ser. 4,5,12:i:-	2 (0.32)	0	1 (1.92)	0	Winter	3 (0.13)
	*Salmonella* Bredeney	0	0	2 (3.85)	1 (2.38)	Winter	3 (0.13)
	*Salmonella* Derby	0	0	1 (1.92)	0	Rainy	1 (0.04)
	*Salmonella* Brancaster	0	0	2 (3.85)	2 (4.76)	Rainy	4 (0.18)
	*Salmonella* Corvallis	0	2 (3.23)	3 (5.77)	3 (7.14)	Rainy	8 (0.36)
	*Salmonella* Singapore	5 (7.94)	7 (11.29)	8 (15.38)	3 (7.14)	Rainy	23 (10.31)
	*Salmonella* Oslo	5 (7.94)	3 (4.84)	0	0	Rainy	8 (0.36)
	*Salmonella* Bovismorbificans	4 (6.35)	6 (9.68)	0	0	Rainy	1 (0.45)
	*Salmonella* Kentucky	1 (1.59)	0	0	0	Rainy	1 (0.04)
C	*Salmonella* Mbandaka	1 (1.59)	4 (6.45)	7 (13.46)	4 (9.52)	Rainy	16 (0.72)
	*Salmonella* Molade	1 (1.59)	2 (3.23)	2 (3.85)	5 (11.9)	Rainy	1 (0.45)
	*Salmonella* Bareilly	1 (1.59)	0	10 (19.23)	7 (16.66)	Winter	18 (0.81)
	*Salmonella* Rissen	1 (1.59)	0	3 (5.77)	5 (11.9)	Winter	9 (0.40)
	*Salmonella* Infantis	0	0	0	1 (2.38)	Winter	1 (0.04)
	*Salmonella* Apeyeme	1 (1.59)	1 (1.61)	3 (5.77)	0	Winter	5 (0.22)
	*Salmonella* Albany	0	0	0	1 (2.38)	Rainy	1 (0.04)
	*Salmonella* Virchow	0	0	0	1 (2.38)	Winter	1 (0.04)
D	*Salmonella* Enteritidis	6 (9.52)	3 (4.84)	0	0	Winter	9 (0.40)
E	*Salmonella* Anatum	0	0	0	1 (2.38)	Rainy	1 (0.04)
I	*Salmonella* Krefeld	1 (1.59)	0	0	0	Summer	1 (0.04)
	*Salmonella* Hvittingfoss	3 (4.76)	3 (4.84)	2 (3.85)	4 (9.52)	Winter	1 (0.54)
Total		63	62	52	42		223

Among positive isolates, *S*. Agona, as the predominant circulating serotype, exhibited significant seasonal variation in prevalence (χ² = 15.64, p < 0.001, Table 6). Its prevalence during winter (37.70%) was markedly higher than that during the rainy season (9.78%) and summer (5.56%). These findings indicate that seasonal variation not only affects the overall prevalence of *Salmonella* but also significantly shapes its serotype composition. The distinct seasonal dominance of specific serotypes indicates clear seasonal patterns within the local poultry production chain. Therefore, it is imperative to implement monitoring and control strategies tailored to these seasonal serotype variations.

**Table 6:** The seasonal variation of *Salmonella* Agona.

Season	Positive	Agona	Non-Agona	Rate (%)	χ²	p-value
Summer	38	2	36	5.56		
Rainy	101	9	92	9.78	15.64	<0.001
Winter	84	23	61	37.70		

Chi-square test (df = 2) was performed on the distribution of *S.* Agona across seasons as primary serotype.

### Antibiotic resistance profile

The prevalence of AMR (to at least one drug) among *Salmonella* isolates varied significantly across seasons (χ² = 7.21, p = 0.027, Table 6). The resistance rate was highest in winter (85.5%), followed by summer (74.4%) and the rainy season (58.4%). Further comparative analysis revealed that, relative to summer, isolates recovered in winter had a significantly higher risk of being drug-resistant (RR = 1.15, 95% CI: 1.00–1.32, p = 0.044). In contrast, the risk in the rainy season was lower but did not reach statistical significance (RR = 0.78, 95% CI: 0.61–1.01, p = 0.058).

**Figure 1:**
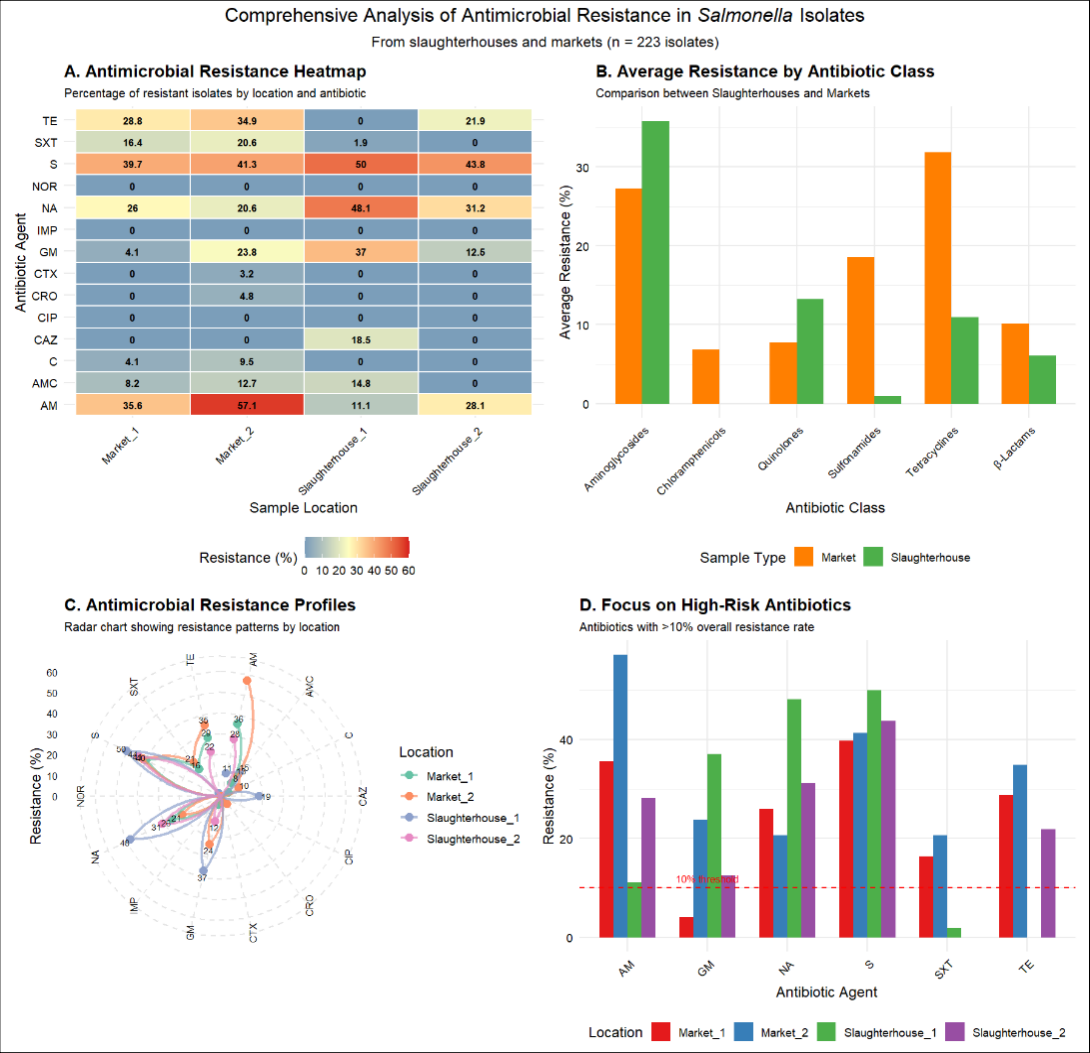
Analysis of antimicrobial resistance in *Salmonella* isolates from slaughterhouse and market sources (n = 223). A. Heatmap: Percentage resistance rates to 14 antimicrobial agents across four sampling locations. Color gradient from green (low) to red (high) indicates resistance levels. B. Bar chart: comparison of average resistance rates by antimicrobial class between slaughterhouses and markets. C. Radar chart: Resistance profiles of the four sampling locations, each line representing one location’s pattern across antimicrobial classes. D. Focused analysis: distribution of high-risk antibiotics (overall resistance >10%) across locations, highlighting Slaughterhouse-1 as a hotspot for gentamicin and nalidixic acid resistance, and Market-2 as a hotspot for ampicillin and tetracycline resistance.

The heat map (Figure 1A) indicates that isolates from all locations exhibited the highest resistance rates to streptomycin (S) (39.7%–50.0%), with particularly pronounced resistance to ampicillin (AM) at Market-2 (57.1%) and Market-1 (35.6%); Slaughterhouse-1 exhibited significantly higher resistance rates to gentamicin (GM, 37.0%) and NA (48.1%) compared to other sites, whereas no resistance was detected to critically important antimicrobials such as CIP, NOR, or IMP. This illustrates severe resistance to first-line drugs, with distinct resistance patterns across locations, suggesting that the emergence and persistence of AMR are closely linked to site-specific environmental factors.

Comparisons revealed that isolates from market sources exhibited higher average resistance rates, particularly to β-lactams and tetracyclines than those from slaughterhouse sources, while isolates from slaughterhouse sources demonstrated higher average resistance rates to aminoglycosides and quinolones (Figure 1B). This indicates distinct antimicrobial selection pressures along the supply chain from slaughterhouses to markets, potentially reflecting variations in antimicrobial usage practices or contamination sources at different stages.

Radar charts revealed unique AMR patterns across locations (Figure 1C), with Slaughterhouse-1 exhibiting the broadest resistance spectrum (the highest levels of multidrug resistance) and Market-2 showing particularly pronounced resistance to certain antimicrobial classes (e.g., β-lactams). These findings confirm significant location-specific resistance profiles, underscoring the need for targeted prevention and control strategies tailored to different settings rather than generic universal approaches.

Analysis of high-risk drugs with overall resistance rates >10% confirmed streptomycin (S), ampicillin (AM), NA, and tetracycline (TE) as primary threats. It clearly showed that Slaughterhouse-1 is the main location contributing to high resistance rates for gentamicin (GM) and NA, while Market-2 is a hotspot for ampicillin (AM) and tetracycline (TE) resistance (Figure 1D). This analysis identifies high-risk antibiotics requiring priority intervention and their core sources, providing direct evidence for implementing targeted surveillance and control measures.

**Figure 2:**
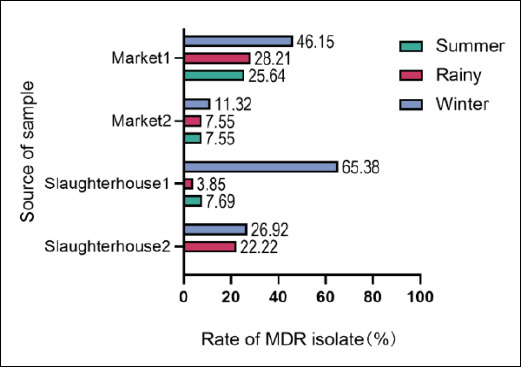
Seasonal and spatial distribution of multidrug-resistant (MDR) *Salmonella* isolates from slaughterhouses and markets (n = 79). Seasonal MDR distribution: Percentage of MDR isolates (resistant to ≥3 antimicrobial classes) across summer, rainy, and winter seasons.

To further investigate the distribution of MDR *Salmonella*, we found that they accounted for 32.43% of total positive cases (n = 79). The prevalence of MDR exhibited significant seasonal patterns, with the highest detection rate occurring in winter (Figure 2). Slaughterhouses (particularly Slaughterhouse-1, with a detection rate of 65.38%) were identified as critical risk points, exhibiting significantly higher levels of resistance and variability compared to market environments (Figure 2). These findings indicate that the slaughter process acts as a key node for the generation and transmission of MDRbacteria, necessitating enhanced targeted surveillance and biosecurity measures during the winter season.

### Genetic diversity and AMR patterns of *Salmonella* isolates

**Figure 3:**
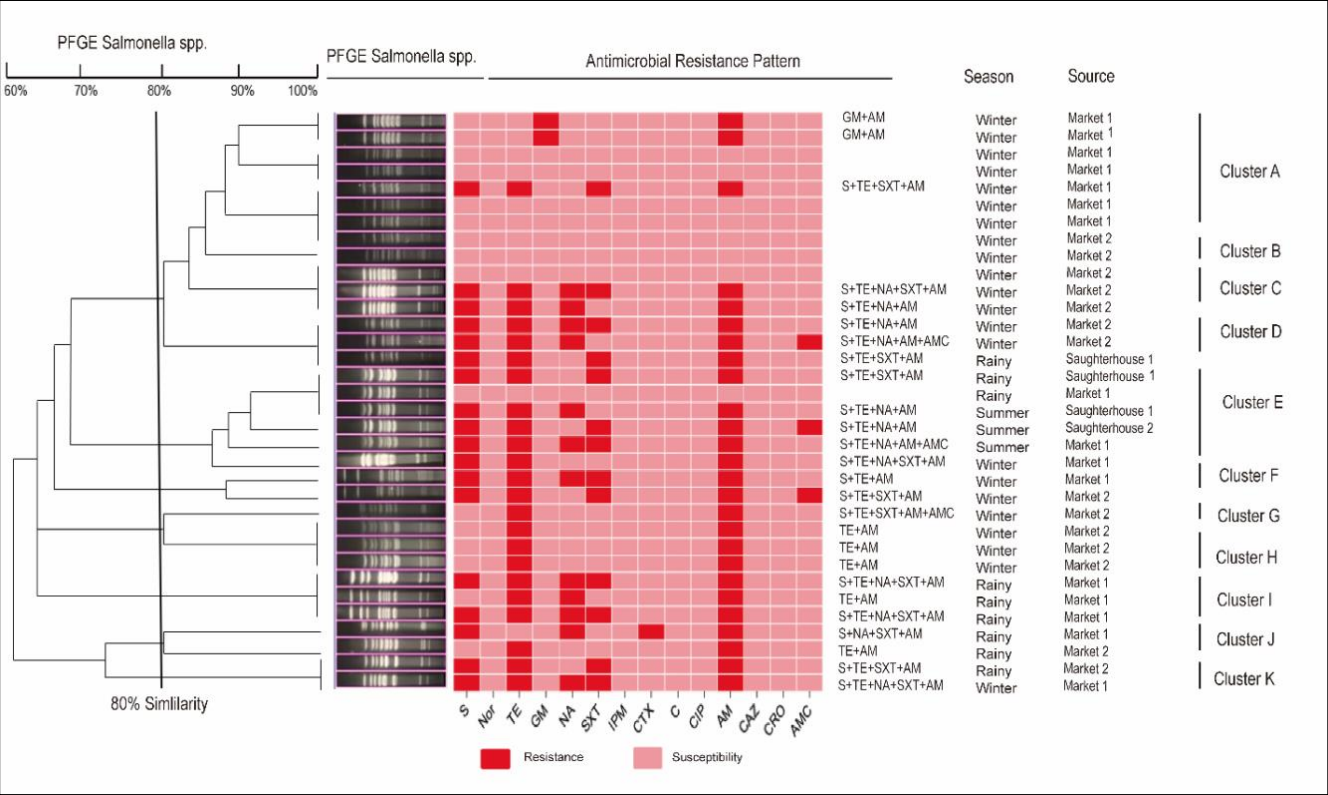
Genetic relatedness of 34 *Salmonella* Agona isolates from slaughterhouses and markets analyzed by pulsed-field gel electrophoresis (PFGE). The clustered using unweighted pair group method with arithmetic mean with Dice similarity coefficient (1% optimization, 1% tolerance). Thirteen distinct PFGE types (A–M) were identified at a 80% similarity threshold.

To explore potential clonal relationships and provide genetic context for the observed spatiotemporal and phenotypic patterns, all available isolates of the most prevalent serotype, *S*. Agona (n = 34), were analyzed by PFGE. These isolates represented the distribution across seasons (summer, rainy, and winter) and sampling locations (Slaughterhouses-1, 2 and Markets-1, 2). Figure 3 presents the resulting dendrogram, corresponding antimicrobial susceptibility patterns, and metadata for the analyzed strains. The isolates were categorized into 13 distinct PFGE types (A-M) using a 78% similarity threshold. In line with the epidemiological data, isolates from the same location and season frequently clustered together, providing molecular evidence for localized clonal persistence or dissemination. Furthermore, the finding that identical PFGE types were shared between some isolates from different locations (e.g., a slaughterhouse and a market) offers genetic evidence consistent with cross-contamination events along the poultry supply chain.

## DISCUSSION

### Overall prevalence and supply chain contamination dynamics

The present study provides a comprehensive overview of the prevalence, serotype distribution, AMR, and genetic relatedness of *Salmonella* isolates recovered from chicken samples originating from slaughterhouses and retail markets in Khon Kaen Province, Thailand, across three distinct climatic seasons. The overall prevalence of *Salmonella* was 32.37%, which is consistent with findings from other developing countries where poultry serves as a major reservoir for this pathogen [24, 25]. The significantly higher contamination rate observed in markets (60.83% in Market 1) compared to slaughterhouses (22.97% in Slaughterhouse 1) underscores the role of post-processing contamination, cross-contamination, and inadequate hygiene practices in retail market environments. These findings align with previous studies conducted in China [26] and Korea [27], where market samples exhibited higher *Salmonella* prevalence than slaughterhouse samples, highlighting the need for enhanced sanitary measures along the poultry supply chain.

### Seasonal variation and environmental drivers

The significant seasonal variation observed, with the highest prevalence during the rainy season (45.29%), parallels findings from tropical regions worldwide [28]. Studies conducted in Bangladesh demonstrated similar seasonal patterns where monsoon conditions facilitated *Salmonella* proliferation in poultry environments [29]. The high temperature (27°C–30°C) and humidity (82%–88%) during Thailand’s rainy season likely create favorable conditions for bacterial survival and spread through water sources and contaminated equipment.

### Seasonal dissociation between prevalence and multidrug resistance

The most novel and operationally relevant finding of this study is the distinct seasonal dissociation between peak *Salmonella* prevalence and peak MDR. While the rainy season (45.29%) posed the highest risk for overall contamination, likely driven by high temperature and humidity that facilitate bacterial survival and cross-contamination [30], the prevalence of MDR peaked significantly in winter, particularly within slaughterhouses (65.38% in Slaughterhouse-1). This dissociation suggests that distinct seasonal mechanisms are responsible: contamination is primarily environmentally mediated, whereas MDR amplification is likely anthropogenic, reflecting seasonal management practices such as prophylactic antibiotic use during colder months or the consolidation of multi-source flocks prior to slaughter [31, 32]. This finding has direct and practical policy implications: rainy season interventions should prioritize hygiene and biosecurity to reduce bacterial load, while winter efforts must focus on antimicrobial stewardship and MDR surveillance within slaughterhouses to prevent the amplification and downstream dissemination of resistant clones.

### Temporal shift in serotype distribution

Comparison with historical data from the same province reveals a notable shift in serotype predominance over the past two decades. While Angkititrakul *et al*. [33] reported *S*. Rissen and *S*. Stanley as the dominant serovars in retail chicken meat in Khon Kaen, our study identified *S*. Agona and *S*. Saintpaul as the predominant serotypes in the local contemporary poultry supply chain. This temporal shift may reflect changes in poultry production systems, biosecurity practices, or antimicrobial usage patterns, and thus warrants further in-depth investigation. The primary contribution of the present study, therefore, lies not in the identification of novel serotypes but in establishing an updated baseline for Northeast Thailand, a region with limited recent epidemiological surveillance data, and documenting, for the first time, the seasonal and spatial distribution patterns of these now-predominant serotypes. Notably, the predominant serotype identified in this study, *S*. Agona, has been consistently reported in earlier human salmonellosis surveillance in Thailand [34], and remains a clinically significant serotype of public health concern. This highlights the need for updated integrated One Health surveillance to assess current transmission dynamics between poultry and human populations.

### Serotype ecology and persistence mechanisms

The predominance of *S*. Agona in slaughterhouses may reflect its well-documented ability to form robust biofilms on abiotic surfaces [35]. For *S*. Saintpaul, genetic determinants involved in surface attachment have been characterized, providing a mechanistic basis for its persistence in processing environments [36]. In contrast, the greater serotype diversity observed in markets, including *S*. Stanley, *S*. Bareilly, and *S*. Rissen, which are historically dominant serotypes, suggests that these serovars continue to circulate in the region, albeit at lower frequencies. This diversity indicates the presence of multiple contamination sources at the retail level, a pattern consistent with recent genomic surveillance in Thai food markets [37].

### AMR patterns and regional context

While direct farm-level antimicrobial usage (AMU) data were not available for correlation with our findings, the resistance patterns observed can be contextualized within the known landscape of AMU in Thai poultry production as reported in the literature. The resistance profiles observed, high rates to streptomycin (43.05%), ampicillin (34.53%), and NA (30.49%), are consistent with national and regional surveillance data from Thailand and neighboring countries [38–40]. The rising resistance gradient from slaughterhouses to markets parallels findings from Vietnam, suggesting additional selective pressure or cross-contamination at the retail level [41].

### Critically important antimicrobials and regulatory impact

The absence of resistance to fluoroquinolones and carbapenems is encouraging and likely reflects Thailand’s 2017 colistin ban and subsequent restrictions on highest-priority critically important antimicrobials in food animals [42]. The notably higher ampicillin resistance in markets (57.14%) compared to slaughterhouses (11.11%) may reflect unauthorized farm-level antibiotic use or cross-contamination during market handling, a pattern observed elsewhere in the region [43].

### MDR dynamics and transmission pathways

The high MDR prevalence in slaughterhouses (65.38% in Slaughterhouse-1), peaking in winter, highlights slaughterhouses as critical nodes for resistance amplification. This seasonal pattern may reflect prophylactic antibiotic use during cold months or flock consolidation prior to winter slaughter [44, 45]. PFGE clustering of isolates by source and season supports localized clonal dissemination, while shared PFGE types across sites suggest potential cross-contamination along the supply chain [46]. These findings reinforce the need for integrated One Health surveillance that links human salmonellosis cases with poultry supply chain data to better inform public health interventions.

### Study limitations and future research directions

Several limitations of the present study should be acknowledged. First and foremost, this study did not collect AMU data from the primary source farms. Consequently, critical information regarding the specific types of antimicrobials used, the distinction between therapeutic and prophylactic applications, and potential seasonal variations in antimicrobial medication practices remains unknown. Without these farm-level AMU records, any robust causal inference between antimicrobial exposure and the observed resistance patterns remains speculative. Second, the absence of comprehensive genotypic characterization of resistance determinants limits our understanding of the underlying resistance mechanisms and their potential for horizontal gene transfer. Third, environmental samples were not collected from sampling sites, restricting our capacity to trace cross-contamination pathways. Fourth, PFGE offers lower discriminatory power than whole-genome sequencing for fine-scale bacterial transmission tracing. Fifth, the cross-sectional design only captures associations at discrete time points but cannot establish temporal causality. Finally, the single-province scope may limit the generalizability of the findings to other regions of Thailand. Despite these limitations, this study provides valuable, much-needed baseline data on the spatiotemporal dynamics of *Salmonella* along the poultry slaughterhouse–market continuum in this understudied region. Future research should systematically integrate longitudinal primary farm-level AMU records, including drug types, purpose, and seasonal patterns, with whole-genome sequencing-based high-resolution molecular surveillance and environmental sampling to definitively elucidate the causal pathways driving AMR emergence and transmission in the Thai poultry sector.

## CONCLUSION

This study provides a comprehensive assessment of *Salmonella* contamination along the poultry slaughterhouse–market continuum in Northeast Thailand, integrating epidemiological, phenotypic, and molecular evidence across seasonal and spatial dimensions. The overall prevalence of *Salmonella* (32.37%) confirms its persistent presence in the poultry supply chain, with significantly higher contamination observed in retail markets compared to slaughterhouses, highlighting the critical role of post-processing handling and hygiene practices. A clear seasonal pattern was identified, with the highest contamination occurring during the rainy season (45.29%), whereas the prevalence of MDR peaked in winter, particularly in slaughterhouse environments. This distinct seasonal dissociation between contamination and resistance represents a key finding of the present study and suggests that different ecological and management-driven mechanisms govern these two processes.

From a practical perspective, these findings provide direct evidence to support targeted, season-specific intervention strategies. Enhanced hygiene, water management, and biosecurity measures should be prioritized during the rainy season to reduce environmental contamination, while winter interventions should focus on antimicrobial stewardship and monitoring of MDR within slaughterhouses to limit the amplification and dissemination of resistant strains. The identification of slaughterhouses as critical nodes for MDR emergence further emphasizes the need for strengthened control measures at this stage of the supply chain.

A major strength of this study lies in its integrated design, combining seasonal surveillance, comparative analysis across supply chain nodes, AMR profiling, and pulsed-field gel electrophoresis-based genetic characteri-zation. This approach enabled the identification of spatial–temporal risk patterns and provided molecular evidence supporting localized clonal dissemination and potential cross-contamination pathways.

Nevertheless, future research should address key limitations by incorporating longitudinal farm-level antimicrobial usage data, environmental sampling, and high-resolution genomic approaches such as whole-genome sequencing to better elucidate transmission dynamics and resistance mechanisms. Expanding surveillance across multiple regions would also improve the generalizability of the findings.

In conclusion, *Salmonella* contamination and MDR dissemination in the poultry supply chain are driven by distinct but interrelated seasonal and spatial factors. The findings underscore the necessity of integrated One Health surveillance and the implementation of targeted, evidence-based control strategies to safeguard food safety and public health.

## DATA AVAILABILITY

Representative original gel images (PFGE) and representative photographs (AST) are provided in the Supplementary Materials. The complete set of original image files is available from the corresponding author upon reasonable request.

## AUTHORS’ CONTRIBUTIONS

ZZ: Investigation, data curation, formal analysis, writing – original draft, and visualization. FS: Methodology, validation, investigation, and writing – review and editing. CP: Resources, software, supervision, and writing – review and editing. SA: Conceptualization, funding acquisition, project administration, supervision, and writing – review and editing. All authors have read and approved the final version of the manuscript.
